# Role of Baseline HIV-1 DNA Level in Highly-Experienced Patients Receiving Raltegravir, Etravirine and Darunavir/Ritonavir Regimen (ANRS139 TRIO Trial)

**DOI:** 10.1371/journal.pone.0053621

**Published:** 2013-01-17

**Authors:** Charlotte Charpentier, Catherine Fagard, Céline Colin, Christine Katlama, Jean-Michel Molina, Christine Jacomet, Benoit Visseaux, Anne-Marie Taburet, Françoise Brun-Vézinet, Geneviève Chêne, Yazdan Yazdanpanah, Diane Descamps, the ANRS139 TRIO Trial study group

**Affiliations:** 1 Laboratoire de Virologie, Assistance Publique-Hôpitaux de Paris (AP-HP), Groupe Hospitalier Bichat-Claude Bernard, HUPNVS, Université Paris Diderot, Paris 7, PRES Sorbonne Paris Cité, EA4409, Paris, France; 2 Université de Bordeaux, ISPED, Centre INSERM U897-Epidémiologie Statistique, Bordeaux, France; 3 INSERM, ISPED, Centre INSERM U897-Epidémiologie Statistique, Bordeaux, France; 4 AP-HP, Hôpital Pitié-Salpêtrière, Service de Maladies Infectieuses, INSERM U943, UPMC, Paris 6, Paris, France; 5 AP-HP, Hôpital Saint-Louis, Service de Maladies Infectieuses, Université Paris-Diderot, Paris 7, Paris, France; 6 Hôpital Gabriel Montpied, Clermont-Ferrand, France; 7 AP-HP, Hôpital Bicêtre, Hôpitaux Universitaires Paris Sud, Pharmacie service de pharmacie clinique, CHU de Bicêtre, AP-HP, Le Kremlin Bicêtre, France; 8 CHU de Bordeaux, Pôle de Santé Publique, Service d’information médicale, Bordeaux, France; 9 AP-HP, Groupe Hospitalier Bichat-Claude Bernard, HUPNVS, Service de Maladies Infectieuses, Université Paris-Diderot, Paris 7, Paris, France; University of Pittsburgh Center for Vaccine Research, United States of America

## Abstract

**Objective:**

In the ANRS 139 TRIO trial, the use of 3 new active drugs (raltegravir, etravirine, and darunavir/ritonavir), resulted in a potent and sustained inhibition of viral replication in multidrug-resistant treatment-experienced patients. The aim of this virological sub-study of the ANRS 139 TRIO trial was to assess: (i) the evolution of HIV-1 DNA over the first year; and (ii) the association between baseline HIV-1 DNA and virological outcome.

**Methods:**

Among the 103 HIV-1-infected patients included in the ANRS-139 TRIO trial, HIV-1 DNA specimens were available for 92, 84, 88, and 83 patients at Week (W)0, W12, W24, and W48, respectively. Quantification of total HIV-1 DNA was performed by using the commercial kit “Generic HIV DNA Cell” (Biocentric, Bandol, France).

**Results:**

Baseline median HIV-1 DNA of patients displaying virological success (n  = 61), viral blip (n  = 20), and virological failure (n  = 11) were 2.34 log_10_ copies/10^6^ PBMC (IQR  = 2.15–2.66), 2.42 (IQR  = 2.12–2.48), and 2.68 (IQR  = 2.46–2.83), respectively. Although not statistically significant, patients exhibiting virological success or viral blip had a tendency to display lower baseline HIV-1 DNA than patients experiencing virological failure (*P*  = 0.06). Median decrease of HIV-1 DNA between baseline and W48 was -0.13 log_10_ copies/10^6^ PBMC (IQR = -0.34 to +0.10), mainly explained by the evolution from W0 to W4. No more changes were observed in the W4-W48 period.

**Conclusions:**

In highly-experienced multidrug-resistant patients, HIV-1 DNA slightly decreased during the first month and then remained stable during the first year of highly potent antiretroviral regimen. In this population, baseline HIV-1 DNA might help to better predict the virological response and to tailor clinical therapeutic management as more aggressive therapeutic choices in patients with higher baseline HIV-1 DNA.

## Introduction

HIV-1 DNA is a major and independent predictor of disease progression in untreated patients with primary or recent HIV infection [1], [2]. Recently, it has been reported that higher baseline HIV-1 DNA was associated with a higher risk of virological rebound in virologically-controlled patients switching their combined antiretroviral-based regimen to a protease inhibitor (PI) monotherapy with darunavir (ANRS-136 Monoï trial) [3]. However, few data are available about the predictive value of baseline HIV-1 DNA on virological response in highly-experienced patients receiving new antiretroviral drug classes such as integrase inhibitors, or new compounds in former classes such as etravirine (ETR) or darunavir (DRV).

A non comparative study, the “Agence nationale de recherches sur le SIDA et les hépatites virales” (ANRS)-139 TRIO trial, showed that a regimen containing three new drugs: the integrase inhibitor raltegravir (RAL), ETR and DRV boosted with ritonavir (DRV/r), resulted in a sustained inhibition of viral replication in multidrug-resistant treatment-experienced patients, with 86% and 88% of patients displaying HIV-1 RNA below 50 copies/mL at one and two years, respectively [4], [5].

The aim of this virological sub-study of the TRIO trial was to assess: (i) the evolution of HIV-1 DNA over the first year; and (ii) the association between baseline HIV-1 DNA and virological outcome.

## Patients and Methods

Among the 103 HIV-1-infected patients included in the ANRS-139 TRIO trial [4], HIV-1 DNA specimens were available for 92, 84, 88, and 83 patients at Week (W)0, W12, W24, and W48, respectively. Peripheral blood mononuclear cells (PBMC) were obtained by Ficoll-Hypaque density gradient centrifugation.

Quantification of total HIV-1 DNA was performed by using the commercial kit “Generic HIV DNA Cell” (Biocentric, Bandol, France).

In the ANRS-139 TRIO trial, virological failure was defined as a plasma HIV-1 RNA level >50 copies/mL at W24; or >50 copies/mL on two consecutive specimens between W24 and W48 for those below 50 copies/mL at W24. A viral blip was defined as an isolated HIV-1 RNA measurement below 400 copies/mL.

HIV-1 DNA levels are described using median and interquartile range (IQR) and comparative analysis between subgroups of patients used non-parametric Kruskal-Wallis test. All statistical analyses were performed using SAS, version 9.1.3 service pack 2 (SAS Institute).

### Ethics Statement

Written informed consent was obtained from all patients. The protocol was reviewed and approved by an ethics committee (Comité de Protection des Personnes) and competent health authorities (Agence Française de Sécurité Sanitaire des Produits de Santé). The trial was conducted in accordance with the Declaration of Helsinki.

## Results

At baseline, HIV-1 DNA was available in 92 patients who did not differ from the 11 other patients of the trial for the main demographic and immuno-virological characteristics (data not shown). At baseline, median HIV-1 DNA was 2.41 log_10_ copies/10^6^PBMC (IQR  = 2.17–2.67). Among these 92 patients, 61 exhibited virological success, 20 experienced a viral blip between W24 and W48, and a virological failure was reported for 11. Most of viral blips were at a low-level viremia (<100 copies/mL in 16 patients).

Baseline median HIV-1 DNA of patients displaying virological success, viral blip, and virological failure were 2.34 log_10_ copies/10^6^PBMC (IQR  = 2.15–2.66), 2.42 (IQR  = 2.12–2.48), and 2.68 (IQR  = 2.46–2.83), respectively. Although not statistically significant, patients exhibiting virological success or viral blip had a tendency to display lower baseline HIV-1 DNA than patients experiencing virological failure (*P*  = 0.06).

**Figure 1:**
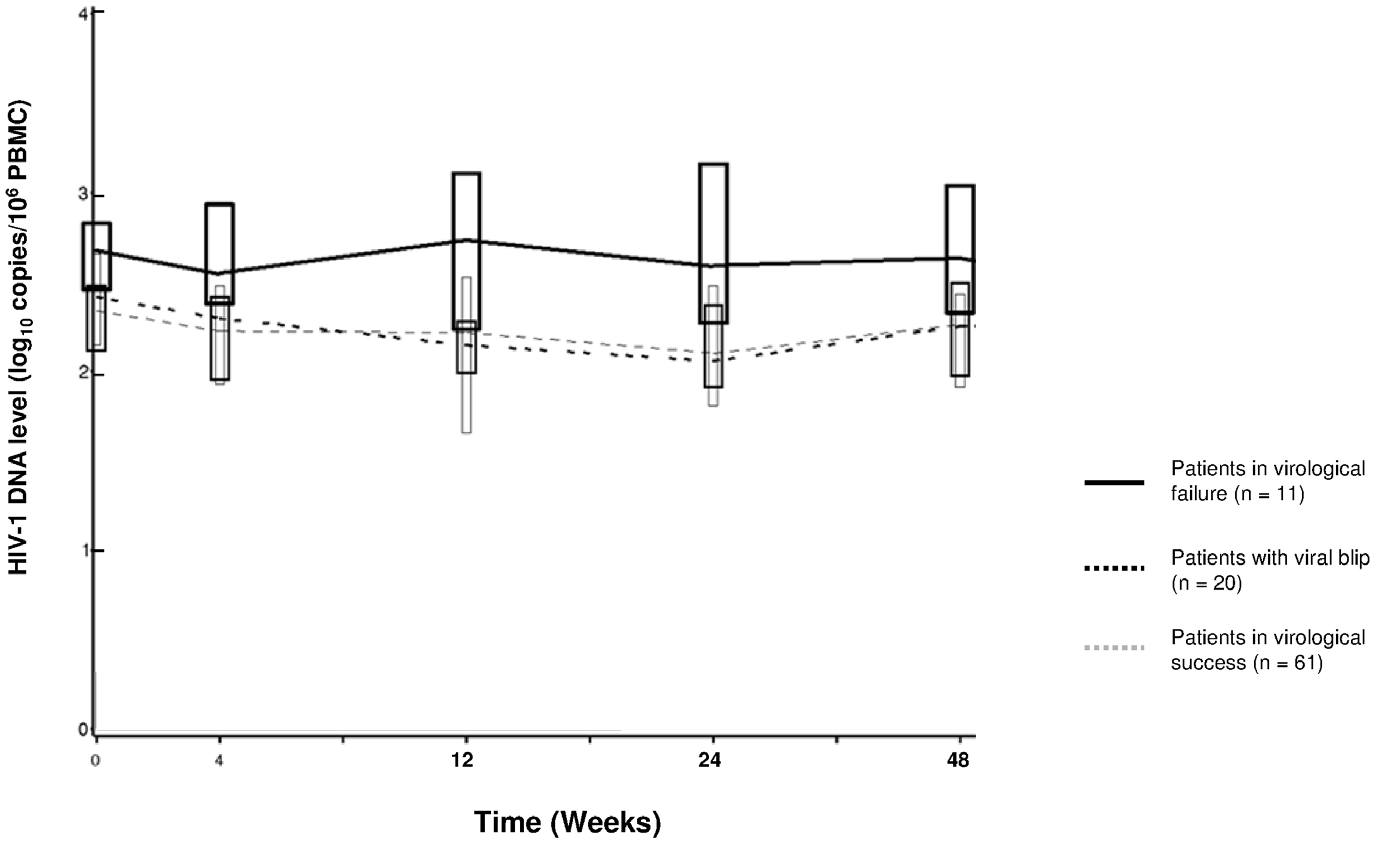
Longitudinal follow-up of HIV-1 DNA level during the ANRS 139 TRIO Trial.

Median decrease of HIV-1 DNA between baseline and W48 was -0.13 log_10_ copies/10^6^ PBMC (IQR = -0.34 to +0.10), mainly explained by the evolution in the first 4 weeks after inclusion with a median HIV-1 DNA of 2.28 log_10_ copies/10^6^PBMC at W4 (IQR  = 1.96–2.52). No more changes were observed in the W4-W48 period (median HIV-1 DNA level  = 2.22 log_10_ copies/10^6^PBMC at W12, 2.16 at W24, 2.26 at W48). Evolution of HIV-1 DNA in patients displaying virological success, viral blip or virological failure is depicted in Figure 1.

## Discussion

In 92 highly-experienced patients receiving 3 active antiretroviral drugs, HIV-1 DNA showed only a modest decrease, mainly observed during the first 4 weeks of the trial. Furthermore, patients exhibiting virological success and/or viral blip had a tendency to display lower baseline HIV-1 DNA than patients experiencing virological failure during the first year of this regimen.

We acknowledge that the main limitation of our analysis is the lack of statistical power to assess the association between baseline HIV-1 DNA level and virological outcome.

In our study, median baseline HIV-1 DNA was 2.41 log_10_ copies/10^6^ PBMC, close to the lower bound of HIV-1 DNA levels previously described in highly-experienced patients [6], [7]. Indeed, in these viremic patients, HIV-1 DNA is reported to be around 3.5 log_10_ copies/10^6^PBMC [6], [7]. Moreover, in our analysis, no significant change of HIV-1 DNA was observed during the 48 weeks of follow-up, as expected and previously described in highly-experienced patients infected for a long time and with a large viral reservoir, where a median decrease of about 0.20 log_10_ copies/10^6^PBMC was reported at 48 or 72 weeks [6], [8], [9]. In other studies, the most important decrease in HIV-1 DNA has been reported in the context of antiretroviral-treated acute infection, i.e. around −1 log_10_ copies/10^6^CD4-T-cells [10].

HIV-1 DNA level is an independent predictor of disease progression in untreated patients with primary HIV infection and during the first 6 months after seroconversion [1], [2]. However, in the population of antiretroviral-experienced patients, HIV-1 DNA is not suggested to be a predictive factor of virological outcome, except in a recent report of PI-monotherapy with DRV/r where higher baseline HIV-1 DNA was associated with a higher risk of virological rebound at W96 [3]. In our study, baseline HIV-1 DNA was only modestly associated with virological failure at one year, though the trend was not statistically significant. Larger studies are needed to confirm that HIV-1 DNA might represent a parameter of interest even in highly-experienced patients with a large size of viral reservoir. Indeed, in viremic highly-experienced patients initiating a new antiretroviral-based therapy, in whom plasma viremia is frequently at low-level, our findings showed that baseline HIV-1 DNA might help to better predict the virological response.

In the present analysis, HIV-1 DNA in patients experiencing a viral blip, mostly below 100 copies/mL, behave closely to the evolution observed in patients experiencing virological success than those in virological failure. As in previous studies assessing the impact of viral blip on viral reservoir size, our findings might suggest that the occurrence of isolated viral blips is unlikely to have the same impact on the replenishment of viral reservoir in virologically-suppressed patients than in viremic highly-experienced patients with a large reservoir size [11], [12].

In conclusion, HIV-1 DNA remains stable during the first year of highly potent antiretroviral regimen in highly-experienced patients. In this population, HIV-1 DNA might nevertheless be a biomarker of potential interest for clinical management if the association of baseline levels with further virological outcome is confirmed in larger studies.
